# The Functions of MicroRNA-200 Family in Ovarian Cancer: Beyond Epithelial-Mesenchymal Transition

**DOI:** 10.3390/ijms18061207

**Published:** 2017-06-06

**Authors:** Pui-Wah Choi, Shu-Wing Ng

**Affiliations:** Division of Gynecologic Oncology, Department of Obstetrics, Gynecology, and Reproductive Biology, Brigham and Women’s Hospital, Harvard Medical School, Boston, MA 02115, USA; puiwahchoi@gmail.com

**Keywords:** ovarian cancer, microRNA-200, epithelial-mesenchymal transition

## Abstract

The majority of studies on microRNA-200 family members (miR-200s) in human cancers are based on the premise that miR-200s maintain epithelial cell integrity by suppressing epithelial-mesenchymal transition (EMT) through direct inhibition of mesenchymal transcription factors zinc finger E-box-binding homeobox 1/2 (ZEB1/ZEB2) and transforming growth factor-β (TGF-β), a potent inducer of EMT. Hence, downregulation of miR-200 in cancer cells promotes EMT and cancer metastasis. Yet, miR-200s are highly expressed in ovarian cancer, and ovarian cancer metastasizes primarily by dissemination within the pelvic cavity. In this review, we will refocus the epithelial property of ovarian cancer cells and the role of miR-200s in safeguarding this property, as well as the diverse roles of miR-200s in inclusion cyst formation, cancer cell growth, collective movement, angiogenesis, exosome-mediated cell communication, and chemoresponse. Taken together, miR-200s play a significant role in the initiation, progression and metastasis of ovarian cancer and may serve as diagnostic biomarkers and a target in therapeutic development.

## 1. Introduction

MicroRNAs (miRNAs) are short single-stranded non-coding RNAs (~22 nucleotides in length) which are able to negatively regulate gene expression by binding to complementary sites in the target messenger RNA (mRNA) at the RNA-induced silencing complex (RISC) and inducing mRNA degradation or translation inhibition [1]. miRNAs have been recognized as molecular regulators and potential therapeutic targets in cancers. miR-200 family is one of the several miRNAs that have been studied for the diagnosis and prognosis of ovarian cancers [2] and also well known as the master suppressor of epithelial-mesenchymal transition (EMT) for the expression of E-cadherin, the classical calcium-dependent cell–cell adhesion protein to maintain epithelial cell phenotype [3]. This miRNA family consists of five members: miR-200a, miR-200b, miR-200c, miR-141 and miR429. These five miRNAs form two clusters: cluster 1 of miR-200b, miR-200a and miR-429 maps to chromosome 1 (1p33.36), while the miR-200c and miR-141 cluster maps to chromosome 12 (12p13.31) [4]. Alternatively, the miR-200 family members can also be categorized in two functional groups based upon the similarities of their seed sequences. miR-200b, miR-200c and miR-429 (Functional Group I) all share the same seed sequence (5′-AAUACUG-3′), while miR-200a and miR-141 (Functional Group II) both share the same seed sequence (5′-AACACUG-3′), with the two functional groups differing in the seed sequence only by one nucleotide [5]. Our analysis of the miR-200 expression levels in ovarian cell lines growing in three-dimensional (3D) cultures suggests that the expression of miR-200s shows cluster-dependent co-regulation [6].

miR-200s are highly conserved among vertebrate species and even in bilaterian animals, with miR-8 being the sole homolog of miR-200 in *Drosophila melanogaster* [7], implying that they possess important functions in a diversity of developmental processes. Table 1 summarizes the results of a myriad of studies, which suggest that miR-200 members play a critical role in the differentiation and proliferation of neurons [8,9,10], podocyte differentiation [11], taste bud formation [12], insulin signaling pathway regulation in the control of fat body and body size [7], and for the hormonal regulation of endometrial stromal decidualization [13,14] during embryo implantation [15]. 

Most of the studies on the miR-200 family in cancer research have been focused on the function of this miRNA family in suppressing EMT, leading to E-cadherin overexpression, epithelial cell identity, and cancer metastasis inhibition [3,18,19]. However, this scenario appears to be different in ovarian cancer, as ovarian cancer cells are more epithelial in nature when compared with their normal counterparts. Cells on the ovarian surface epithelium and the associated cortical inclusion cysts have been suggested as the origin for ovarian cancer development [20,21]. Incessant ovulation hypothesis states that consistent damage to ovarian surface epithelial (OSE) cells results in Müllerian metaplasia and tumorigenesis [20,22]. Several mouse models have shown that ovarian tumors originated from ovarian surface epithelia [23,24,25,26]. The laying hen animal model for spontaneous ovarian cancer development [27,28] is consistent with the incessant ovulation hypothesis, since ovulation arrest resulted in lower ovarian cancer incidence [29,30,31]. Recent studies, however, have suggested that epithelial cells in the distal fallopian tube can be the cells of origin for the development of serous subtype of ovarian cancer. Serous tubal intraepithelial carcinomas (STIC) with *p53* mutations that are frequently observed in high-grade serous ovarian carcinoma (HGSOC) are proposed as a potential precursor [32,33,34,35]. Both ovarian coelomic epithelial cells and fallopian tube Müllerian epithelial cells possess mesenchymal characteristics [20,36], suggesting a different EMT scenario for miR-200 involvement. In addition, spatial and temporal presence or absence of the miRNA and putative targets may also play a significant role. Herein we review the function of miR-200 on both pathogenesis and other aspects of ovarian cancer, and the potential utility in clinical diagnosis and therapies.

## 2. miR-200 and Ovarian Cancer

### 2.1. miR-200, EMT, and Epithelial Nature of Ovarian Cancer Cells

miR-200s are well known to have a role in controlling EMT in various types of cancer, including breast cancer, pancreatic cancer, colorectal cancer [37], prostate cancer [38], and ovarian cancer [39,40]. Indeed, across NCI60 cell lines, expression of miR-200s is correlated with the epithelial phenotype. This implies miR-200s is a universal indicator of epithelial phenotype of cancer cells. In clinical samples, sarcomatoid metaplastic breast tumor cells have lower expression of miR-200s when compared to the more epithelial-like ductal breast cacinomas. [19]. The recent Cancer Genome Atlas (TCGA) genomic study of uterine carcinomasarcomas has also shown that sarcomas and uterine carcinosarcomas with extensive sarcoma components showed enhanced promoter methylation and down-expression of miR-200 family [41]. In ovarian tissue, miR-200s are generally up-regulated in ovarian tumors compared with normal ovarian tissue [42,43,44], with variable expression levels according to histological subtypes and stages of ovarian cancer. We have also shown by quantitative reverse transcription-polymerase chain reaction (RT-PCR) that miR-200 pathway genes are up-regulated in microdissected cells derived from ovarian tumor tissues and serous tubal intraepithelial carcinoma (STIC), a precursor lesion for high-grade serous ovarian carcinoma (HGSOC) [32,33,45,46,47], relative to normal ovarian surface epithelial (OSE) and fallopian tube epithelial (FTE) cells. In accordance with these results, malignant ovarian tumors were found to express more epithelial markers than normal or benign ovaries [6,20,21,36,48,49]. 

We and others have shown that the epithelial characteristics of ovarian cancer cells are indeed very important for the growth and survival of the epithelial cancer cells. The pioneer work performed by Auerspeg et al. [50] on E-cadherin suggested that increased E-cadherin expression in ovarian surface epithelium induced mesenchymal-epithelial-transition (MET), and the resulting cells formed tumors in immunodeficient mice [51]. Increased expression of E-cadherin was found in surface invaginations and particularly in epithelial inclusion cysts, which are sites of frequent metaplastic and dysplastic changes. Moreover, E-cadherin mRNA and protein expression were also significantly upregulated in the ovarian adenocarcinomas of the laying hen preclinical model for spontaneous ovarian cancer apparently due to incessant ovulations [27], suggesting that up-regulation of E-cadherin is an early defining event in ovarian cancer development [27]. E-cadherin/β-catenin complex can activate PI3K/AKT pathway [52] and epidermal growth factor receptor (EGFR) for the survival and proliferation of cancer cells [53]. E-cadherin was also reported to promote proliferation of human ovarian cancer cells in vitro by activating MEK/ERK pathway [54]. Furthermore, our work has shown that nuclear phosphoprotein Pinin and some epigenetic transcriptional co-repressor proteins play key roles in epithelial cell identity, growth and multidimensional adhesion of ovarian cancer cells [55]. A recent characterization of a mouse model of HGSOC originating in the fallopian tube stroma showed epithelialization of the stromal cancer cells to support tumorigenesis [56]. Interestingly, increased expression of miR-200 and E-cadherin was also identified in the laying hen model of spontaneous ovarian carcinoma [27,28], implicating the importance of this conserved pathway in ovarian cancer development. Taken together, the results of all these studies support the notion that epithelial characteristics of ovarian cancer cells are important for the growth and propagation of epithelial ovarian cancer.

miR-200s, as the master regulators of epithelial cell identity, play an important role in controlling the epithelial characteristics and the growth of cancer cells. Regulation of miR-200 family on EMT and E-cadherin expression is well documented [2,3,18,19]. Specifically, in growth, miR-200s were shown to activate PI3K/AKT by targeting FOG2 (friend of GATA 2), which directly binds to the p85α regulatory subunit of PI3K [57]. In lung cancer, miR-200s increase AKT activity in a FOG2-independent manner by concomitantly inactivating S6K and increasing the level of IRS-1, a S6K substrate. Depletion of IRS-1 partially inhibited the miR-200-dependent AKT activation [57].

### 2.2. miR-200, Collective Movement, and Ovarian Cancer Metastasis

The conventional framework for tumor invasion and dissemination is EMT, the mechanism by which cancer cells migrate as elongated fibroblast-like single cells with the help of proteinases to breakdown the extracellular matrix [58]. However, many cancer invasion studies [59,60] have shown that cancer cells can also migrate as interconnected cell groups, termed collective movements, as observed in morphogenetic cell movements during tissue remodeling and organ development [61,62]. Most of the studies about miR-200s address the role of miR-200s on inhibition of cancer cell metastasis by miR-200s via EMT. Two key molecular targets in the miR-200s-mediated EMT pathway are the receptor-regulated *SMADs* (*SMAD2* and *SMAD3*) and *ZEB* family mRNAs (*ZEB1* and *ZEB2*). *ZEB2* has two predicted miR-200a/141 and five miR-200b/200c/429 seed matches in its 3′ untranslated region (3′-UTR). *ZEB1* carries five putative miR-200b/200c/429 and three miR-200a/141 sites in its 3′-UTR [3]. 3′-UTR of *TGF-β2* contains a target site for miR-141/200a [63,64]. Similar to mothers against decapentaplegic family member 2 and 3 proteins (SMAD2 and SMAD3) are the downstream effectors of TGF-β signaling pathway, whereby the activation of the TGF-β receptor leads to phosphorylation of SMAD2 and SMAD3. Activated SMAD2 and SMAD3 will bind to SMAD4 and induce translocation to the nucleus, in which they will interact with different nuclear regulators and modulate transcription of target genes and induce a mesenchymal cell phenotype [65]. ZEB1 and ZEB2 are zinc finger proteins which regulate expression of multiple genes. ZEB1 and ZEB2 are transcriptional suppressors of E-cadherin [3]. Besides, vimentin promoter can be activated by ZEB2 by an unknown mechanism [66]. Autocrine TGF-β signaling is required for the maintenance of a stable mesenchymal phenotype to drive sustained ZEB expression [67]. Besides targeting *ZEB1*/*ZEB2* to up-regulate E-cadherin expression, miR-200s also target *Snail* to increase E-cadherin expression in ovarian cancer [68]. Application of miR-200 antagomirs can reverse EMT caused by the depletion of ERRα molecules, with an increase of *Snail* expression [68]. Many in vitro studies have strongly supported that miR-200s expression determines cellular expression of epithelial or mesenchymal biomarkers in endometrial [69] and ovarian cancer cell lines [40]. The resulting cells display distinct MET or EMT phenotypes [70]. However, studies of clinical samples are not conclusive to relate the expression level of miR-200 family with disease stage. A study to analyze miR-200 expression in tissue from patients with or without relapse showed that miR-200b and miR-200c were down-regulated in relapsers compared with non-relapsers. Multivariate analysis confirmed that down-regulation of miR-200c in the test set was associated with overall survival, independent of clinical covariates [71]. Conversely, Cao et al. reported that elevated expression of miR-200a and miR-200b was associated with advanced stage and high-grade tumors, while high miR-200c expression was only associated with advanced tumors [72].

Unlike other human cancers, ovarian cancer rarely disseminates through the vasculature [73]. Ovarian cancer metastasizes primarily via direct extension or detachment from the primary tumors. Tumor cells are also carried passively by the peritoneal fluid or ascites fluid as cancer cell spheroids and disseminate within the peritoneal cavity [73]. Iwanicki et al. have employed time-lapse video microscopy to elegantly illustrate how tumor spheroids use integrin-mediated activation of myosin to produce traction force for replacing mesothelial cells as a step for tumor implantation on the walls of peritoneal and pleural cavity [74]. We have employed sequential in vitro three-dimensional (3D) cultures to study the movement of cancer spheroids in collagen I extracellular matrix [6]. The cancer cells showed elevated expressions of E-cadherin and migrated in an interconnecting mass resembling the collective movement described for tissue remodeling and in some cancer invasion studies [59,60,61]. Gene expression analysis of the 3D cultures and clinical ovarian samples showed concordant increased expression of E-cadherin and miR-200s, but suppressed expression of TGF-β pathway genes in migrating 3D tumor spheroids and tissues than in normal OSE spheroids and ovarian tissues. Perturbation of this pathway by knocking down E-cadherin expression in ovarian cancer cells via RNA interference suppressed the 3D collective movement of cancer cell spheroids [6]. Wyckoff et al. have identified the ROCK-myosin signaling axis as the mechanism by which tumor cells invade collectively through 3D matrices in a protease-independent manner [75]. Hence, miR-200s are not only important for the epithelial phenotype necessary for ovarian cancer cell growth, but apparently also play an important role for the ROCK-myosin regulation during collective movement and local metastasis of ovarian tumors.

In our sequential in vitro 3D model in collagen I matrix, we noted that migrating HGSOC spheroids tended to release some single cells in the later phase of migration [6], and quantitative real-time PCR also demonstrated that HGSOC samples had a lower level of expression of miR-200 pathway genes compared to other histologic subtypes of ovarian tumors. Pradeep et al. have reported an interesting study, in which two mice were surgically anastomosed from the shoulder to the hip joint to form a shared vasculature between the pair of mice [76]. One mouse was injected with tumor cells directly into the ovary and the parabionts were separated two weeks following the tumor cell inoculation. Tumors were found first in the omentum of the guest mouse on day 25 before spreading to other organs later, suggesting that ovarian cancer cells were transferred hematogenously for omental metastasis [76]. Characterization of circulating tumor cells in the vasculature demonstrated activation of the Erb-B2 receptor tyrosine kinase 3/neuregulin (ErbB3/NRG1) axis and EMT promoted this hematogenous metastasis, and perturbation of ErbB3 signaling inhibited omental metastasis. The results of this interesting study suggest that there can be the presence of an alternative hematogenous omental metastasis besides the classical theory of direct spread of migrating cancer cells. Intriguingly, another study by Zhang et al. showed an opposite function of miR-200 target ZEB1 in *EGFR*-mutated lung cancer cells. Whereas ZEB1 promoted growth in *KRAS*-mutated lung cancer cells, ZEB1 expression suppressed cell growth in *EGFR*-mutated lung cancer cells. Ectopic expression of ZEB1 in *EGFR*-mutated lung cancer cells inhibited cell growth by increasing miR-200c target Notch1, which repressed *ErbB3* promoter activity and the expression of *ErbB3* [77]. As ErbB3 is essential for growth of *EGFR*-mutated lung cancer cells, the ZEB1/miR-200 negative feedback loop might potentially provide a cell context-dependent regulation of ErbB3. Furthermore, a study to characterize the function of an epithelial cell transcription factor implicated in cancer progression, grainhead-like 2 (GRHL2) in ovarian cancer showed that GRHL2 bound to *miR-200* promoters, intronic enhancer of *CDH1*, and promoters of *ErbB3* and other epithelial cell-related genes and positively regulated their expression [78]. Further studies have shown that GRHL2 and ZEB1 formed a double negative feedback loop for the reciprocal regulation of miR-200s expression and epithelial phenotype [78]. Given that all these studies provide seemingly contradictory findings, it is crucial to evaluate all genetic interactions in a cell context-dependent manner in different ovarian cancer cell populations in order to infer the likely metastatic pathways. 

**Figure 1:**
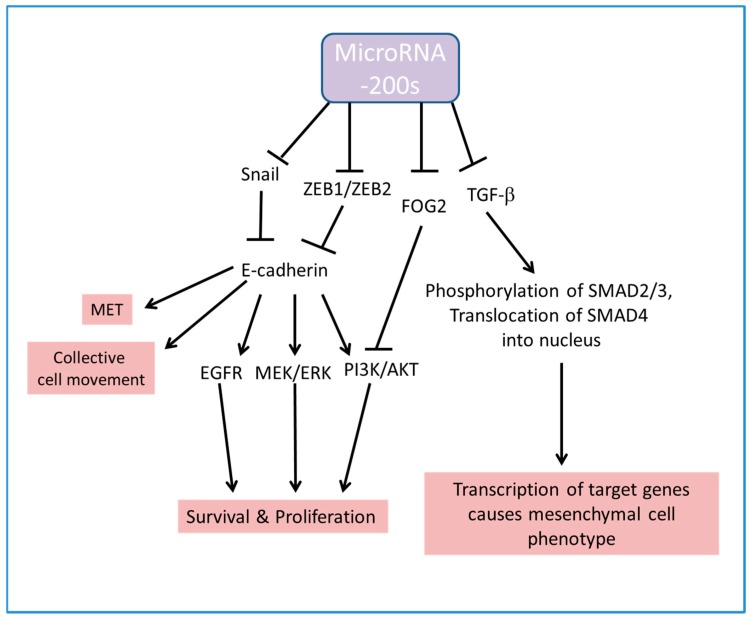
Schematic diagram showing miR-200s target E-cadherin and TGF-β for tight regulation on mesenchymal-epithelial-transition (MET), cell migration and cell survival.

The pathways which are regulated by miR-200s and affect the growth and the epithelial or mesenchymal features of the cells are summarized in Figure 1.

### 2.3. Other Functions of miR-200s in Ovarian Cancer

#### 2.3.1. Inclusion Cyst Formation

Several hypotheses have been put forward to explain the mechanism for development of the epithelial ovarian cancer, with incessant ovulation as one of the important factors contributing to tumorigenesis [79]. Incessant ovulation may cause consistent damage of ovarian surface epithelium through the production of inflammatory factors and reactive oxidants [80]. The extracellular matrix (ECM) proteins, proteases, secreted growth factors and chemokines from the stromal microenvironment can provide oncogenic signals in a paracrine fashion. One likely consequence emerging from this hypothesis is that chronic ovulations cause invaginations of OSE cells, leading to the formation of inclusion cysts that serve as precursors for metaplastic and dysplastic changes leading to cell transformation [81]. In line with the incessant ovulation hypothesis, the number of ovulatory cycles and the risk of getting ovarian cancer is positively correlated [82]. Moreover, multiparity [83], length of lactation [84], and oral contraceptive usage [85] all reduce the risk of getting ovarian cancer. In contrast, many epidemiological studies have shown that a low number of pregnancy and infertility and hence a larger number of ovulatory cycles are linked to the increased incidence of ovarian cancer [22]. Data from animal studies also support the incessant ovulation hypothesis [86].

There is no direct evidence on linking miR-200s to ovarian inclusion cyst formation; several lines of emerging evidence suggest that the miR-200 family plays an important role in renal tubule development, renal cyst pathogenesis and spermatogenic cyst formation in yellow catfish. First, the expression of miR-200 family members is reduced in injured kidney tubules, but highly enriched in the normal kidney tubules. Second, kidney tubule-specific knockout of Dicer, the miRNA biogenesis enzyme, led to significant down-regulation in the expression of all five members of the miR-200 family and formation of kidney tubule-derived cysts [16]. Third, miR-200 knockdown in cultured renal epithelial cells inhibited tubulogenesis and produced cyst-like structures. Taken together, these results implicate that miR-200s have a role in the maintenance of normal renal tubule structure and prevention of cyst formation. Two evolutionary-conserved binding sites for the miR-200 members were identified in the 3′-UTR of the polycystin 1 (PKD1) gene. miR-200s directly bind to PKD1 3′-UTR and inhibit its translation. On the other hand, the transcription of miR-200s is regulated by another cystic kidney disease related gene, *Hnf-1.* Mutations of HNF1β produce cystic kidney disease [87]. In mice, kidney tubule-specific deletion of *Hnf-1β* resulted in decreased expression of miR-200s and caused renal cysts. HNF-1β binds to a promoter region upstream of the miR-200 genes and directly controls the transcription of the miR-200a/b/429 cluster. The regulation on cyst and tubule formation in the kidney by miR-200s is closely related to the EMT function of miR-200s. Renal tubule epithelia in kidneys of *Dicer* and *Hnf-1β* mutant mice undergo partial EMT wherein they simultaneously express both epithelial and mesenchymal markers [16]. The expression of miR-200 targets, ZEB2 and TGF-β2, were increased several folds, without any change in the expression of epithelial protein E-cadherin [87]. While the role of partial EMT in aggravating cyst growth currently remains uncharacterized, partial EMT of renal tubule epithelia has been recently shown to promote renal tubule injury and kidney fibrosis [88].

In yellow catfish, miR-200s are related to the size of spermatogenic cyst. YY testis (super-males), which has a higher degree of testis maturity, had relatively lower expression of miR-141 and miR-429 when compared with XY testis. The more mature YY testis is featured by a larger spermatogenic cyst, more spermatids and fewer spermatocytes in the spermatogenic cyst. In both yellow catfish and human, down-regulation of miR-141 and 429 was observed during the progression of testis development [17].

In human ovary, miR-200 may also have a role in ovarian cyst formation. Our lab used in vitro 3D culture systems to analyze the expression of miR-200s in the cysts formed by normal OSE cells and ovarian cancer cells [6]. Concurrent down-regulation of TGF-β pathway genes and high levels of E-cadherin and miR-200 expression were revealed in the cancerous cysts by gene expression profiling. Transient silencing of E-cadherin expression in ovarian cancer cells disrupted cyst structure formation [6]. More mechanistic studies will provide further insights in this direction. 

The laying hen model is a great surrogate model for preclinical studies of human ovarian cancer. In line with the incessant ovulation hypothesis for the damages to OSE in causing ovarian cancer development, a hen which ovulates daily, on average, would have produced 300 to 500 eggs on her second year of laying, the same number as a woman who has undergone menopause, when the risk of ovarian cancer increases dramatically [89]. Two landmark large-scale epidemiological studies have shown that spontaneous ovarian cancer was first detected in hens aged between 2 and 2.5 years, and the incidence reached a peak of nearly 60% when the hens reach the age of four [90,91]. When ovulation is arrested in hens either by dosing with progestins [29], nutrient deprivation [30], or via genetic selection for low-egg laying variants [31], the incidence of ovarian cancer is significantly lower, further supporting the incessant ovulation hypothesis in spontaneous ovarian cancer development. Hales et al. reported expression of miR-200a, miR-200b and miR-429 were significantly up-regulated in ovarian tumors compared to normal ovaries [28]. As there are also cysts present in pre-malignant ovaries of laying hens, it would be of great interest to study the expression level of miR-200s in the inclusion cysts of both human and laying hen specimens and compare the level from the epithelium of the normal ovary, which may provide a clinical validation of our in vitro 3D results.

#### 2.3.2. Angiogenesis

A previous study on Ishikawa cells (endometrial adenocarcinoma) revealed that restoration of miR-200c expression resulted in reduced mRNA expression and protein level of vascular-endothelial-growth factor (VEGF) [92]. This suppression was attributed to the direct binding of miR-200c to the *VEGF* mRNA 3′-UTR region. Concordantly, a recent study also showed that miR-200c negatively regulated *VEGF* mRNA and protein levels in placenta tissue [93]. Similarly, in lung cancer [94], miR-200c directly targeted VEGF receptor 2. In a bone cancer study, overexpression of miR-200c transformed the metastatic fast-growing tumor into a dormant tumor both in vivo and ex vivo. The survival rate of the mice injected with the tumors was prolonged due to the suppression of angiogenesis. This further suggests that miR-200c negatively regulates angiogenesis. In ovarian cancer, overexpression of miR-200c in an ovarian cancer cell line (Hey8A) decreased IL8 and CXCL1 expressions. This suppression was reversed by the presence of miR-200c inhibitors [95]. IL8 and CXCL1 are critical players of tumor vasculature and angiogenesis [96]. 

**Figure 2:**
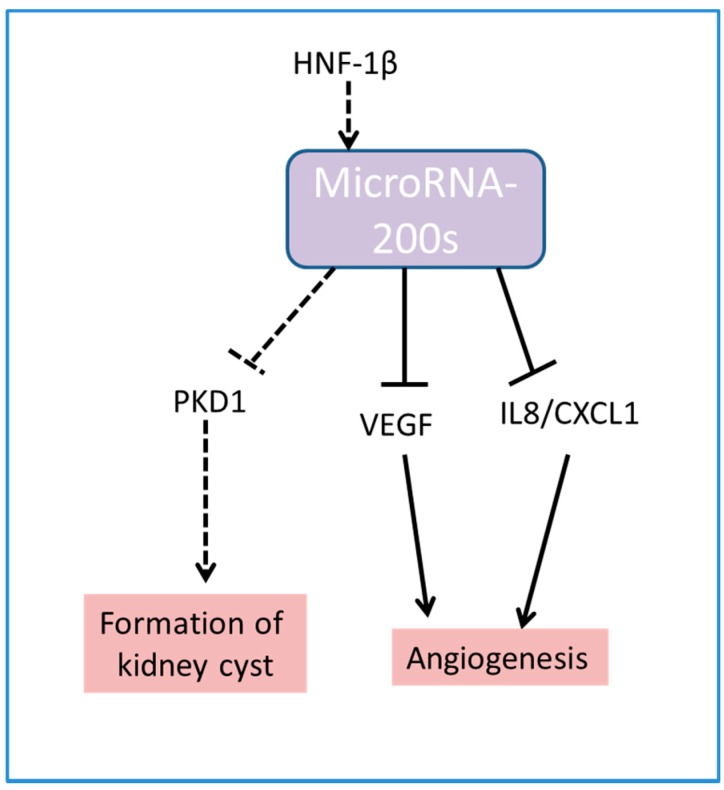
Schematic diagram showing miR-200s control cyst formation and angiogenesis. Dashed lines indicate the same pathway. HNF-1β: hepatocyte nuclear factors 1β, PKD1: polycystin I, VEGF: vascular-endothelial-growth factor, IL8: interleukin 8, CXCL1: chemokine (C-X-C motif) ligand 1.

Figure 2 summarizes the pathways controlled by miR-200s which affect formation of cyst in kidney and angiogenesis.

#### 2.3.3. Exosomes

Exosomes are actively secreted from living tumor cells and are distinct from apoptotic cell-derived microvesicles [97]. Exosomes might play a role in the pathogenesis and metastatic spread of cancer based on the studies which support the notion that the released exosomes modify local extracellular conditions to promote cell growth and neovascularization and may regulate the phenotype of parent and/or targeted cells [98,99]. For examples, exosomes from glioma cells contain selective packaged miRNA that modify the gene expression in the recipient cells [100].

RNAs are not randomly loaded into exosomes. miRNAs can be uploaded into exosomes based on specific ‘shuttle’ sequences. SUMOylated heterogeneous nuclear ribonucleoprotein A2B1 (hnRNPA2B1) specifically binds to miRNAs containing the ‘shuttling’ motif GGAG, which leads to their upload into exosomes. miRNAs that have a high level of expression while their cognate target mRNAs have a low level of expression are more likely enriched in exosomes [101]. In addition, AGO2, a protein associated with the RNA-induced silenced complex (RISC) complex, is thought to control the loading of miRNAs into exosomes [102]. Tumor cells constitutively secrete exosomes, which can perform an important role in the modulation of the host immune response [103], induction of angiogenesis [104], and cell invasion and metastasis [105].

In ovarian cancer, Taylor et al. reported that exosomal miR-200 levels were more elevated in sera from ovarian cancer patients than samples from benign controls [106]. In another study, Kobayashi et al. compared the abundance of miR-200 in exosomes produced by two cell lines with different invasive capacities. The more invasive SKOV-3 cells released 2.7-fold more exosomes when compared to the less invasive OVCAR-3 cells, and miR-200s transcripts were only identified in OVCAR-3 cells and their exosomes [107]. With the involvement of only two cell lines, Kobayashi’s study is not conclusive to associate the relationship between ovarian cancer cell invasiveness with miR-200 contents in the secreted exosomes. Further studies with more cell lines and co-culture experiments are required to provide insight into whether exosomal miR-200 transcript levels are associated with ovarian cancer development. 

#### 2.3.4. Chemoresponse

Currently, the standard of care for the treatment of ovarian cancer is a combination of taxane and platinum chemotherapy, with only modest improvement in outcomes over the last decade. The 5-year survival rate for women with advanced stage disease is only about 30%. More than 50% of patients who initially responded to the treatment relapsed within 18 to 24 months [108] and usually accompanied with chemoresistance.

miR-200 was reported to associate with chemoresponse in various types of cancer [109]. Increased expression of E-cadherin through ZEB1 repression by miR-200b was associated with increased expression of pro-apoptotic genes in the p53 apoptotic pathway and re-sensitized the cancer cells to doxorubicin in breast cancer [110]. Moreover, Sun et al. reported that miR-200b and miR-15b were significantly down-regulated in the chemoresistant tongue squamous cell carcinoma [111]. Ectopic expression of miR-200b and miR-15b with miRNA mimics effectively sensitized the tongue squamous cells to chemotherapy. Such an effect was suggested to act through BMI1, a ring finger protein that is the major component of the polycomb group complex 1, which mediates gene silencing by regulating chromatin structure [112]. miR-200c and miR-141, but not miR-200b, were reported to be associated with chemosensitivity in ovarian cancer. Class III β-tubulin gene (TUBB3) is a classical protein that contributes to cellular resistance to taxane by enhancing the dynamic instability of microtubules, thereby counteracting the activity of microtubule-targeting agents [113]. *TUBB3* is not expressed in normal epithelial cells, but is often overexpressed in taxane-resistant carcinomas and cancer cell lines [113,114]. *TUBB3* is a direct target of miR-200c. In a study reported by Cittelly et al., low miR-200c expression correlated with poor prognosis in ovarian tumors. Restoration of miR-200c expression in an intraperitoneal xenograft model of human ovarian cancer reduced *TUBB3* expression and resulted in a significantly decreased tumor burden [115]. On the contrary, Prilei et al. reported a direct correlation between miR-200c expression and chemoresistance in a panel of ovarian adenocarcinoma cell lines [116]. Analysis of *TUBB3* 3′-UTR-associated complexes identified that miR-200c increased the association of the RNA binding protein HuR, which enhanced *TUBB3* mRNA translation. Also, in the analysis of 220 ovarian cancer samples, the relationship of miR-200c expression with clinical outcome depended on the cellular localization of HuR. When HuR was nuclear, high expression of miR-200c inhibited *TUBB3* expression and resulted in a good prognosis, whereas when HuR occurred in cytoplasm, the same miRNA enhanced *TUBB3* expression and produced a poor outcome [116]. This interesting study suggests a model for the combined regulatory activity of miR-200c and HuR on *TUBB3* expression in ovarian cancer that may not happen in other cell types and provides additional insight into studying potential player(s) that may affect the outcome of miR-200 interaction with target genes.

Besides Taxol, miR-200c and miR-141 also regulate sensitivity to carboplatin. In a study with two ovarian cancer cell lines, OVCAR-3 and MES-OV, and their paclitaxel resistant variants OVCAR-3/TP and MES-OV/TP, resistant variants OVCAR-3/TP showed a marked decrease in miR-200c and miR-141. Resistance to paclitaxel and carboplatin was induced when inhibitors of miR-200c or miR-141 were transfected into the parental OVCAR-3. Notably, both OVCAR-3/TP and MES-OV/TP cells regained sensitivity to carboplatin but only MES-OV/TP regained sensitivity to taxol [117]. Introduction of miR-200c and miR-141 mimics into OVCAR-4/TP resulted in alternation in expression of genes related to oxidative stress. *ALDH1A3*, *TXNDC12*, *RRM2* and *MTHFD2* were up-regulated while *AKR1C1*, *AKRIC4*, and *SCD* were down-regulated. [117].

As mentioned above, oxidative stress is another factor that affects chemoresistance. Accumulation of reactive oxygen species (ROS) contributes to cell damages and alterations in gene expression, proliferation and genomic stability in tumor cells [118]. The p38α mitogen-activated protein kinase (MAPK) family possesses a redox-sensing function which is important in controlling tumor development. [119]. By blocking proliferation or promoting apoptosis, p38α suppresses tumorigenesis [120,121]. miR-141 and miR-200a inhibit protein expression of p38α. Accumulation of miR-141 and miR-200a mimicked p38α deficiency and promoted malignancy in mouse models. Consistent with the finding from the mouse model, human ovarian cancer with high miR-200a expression showed low amounts of p38α protein and an associated oxidative stress signature. Although tumorigenesis is promoted by overexpression of miR-200a or miR-141, overexpression of miR-200a and miR-141 also increases tumor-cell death and slows tumor growth under treatment with paclitaxel, a chemotherapeutic drug known to increase ROS. The miR-200a dependent stress signature is correlated with longer progression-free survival and improved survival of patients in response to chemotherapeutic treatment [122]. Despite increase in tumor growth, the miR-200-dependent stress response could also enhance sensitivity to chemotherapy, indicating its dual function in tumors.

**Figure 3:**
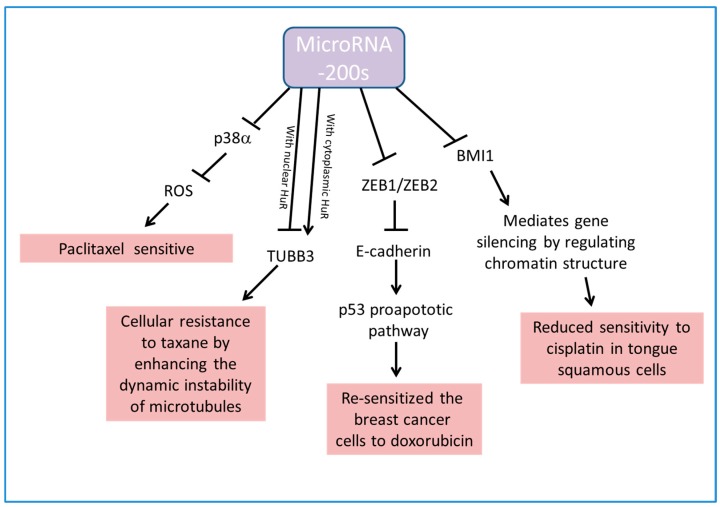
Schematic diagram showing that miR-200s regulate chemoresponse in cancer cells through p38α, TUBB3, E-cadherin and BMI1. TUBB3: Class III β-tubulin gene, ZEB1/2: zinc finger E-box-binding homeobox 1/2, BMI1: polycomb group RING finger protein 4.

Figure 3 summarizes the involvement of miR-200s in generating chemoresponses in several kinds of cancer.

The other functions of miR-200s in ovarian and gynecologic cancers are summarized in Table 2.

### 2.4. Utility of miR-200s in Diagnosis, Prognosis, and Therapeutics of Ovarian Cancer

As miR-200s are highly expressed in epithelial ovarian cancer and are involved in tumorigenesis, progression, metastasis and chemoresponse of the disease, it is rational to pursue whether miR-200s have any clinical utilities. Zuberi et al. measured the level of miR-200a, miR-200b and miR-200c in serum samples of 70 cases of epithelial ovarian cancer with different histological types. The levels of miR-200a in cancer sera samples were found to be six-fold, while the levels of miR-200b and miR-200c were three-fold higher than the levels in normal controls. The expression levels of miR-200a and miR-200c were significantly associated with disease progression. miR-200a overexpression was found to be associated with mucinous histology and advanced stage tumors, and patients with lymph node metastasis showed significant elevation of miR-200c [123]. As mentioned in the Exosome section, Taylor et al. identified elevated exosomal miR-200 levels in the sera of ovarian cancer patients than in benign controls, and the circulating miRNA profiles accurately reflected the miRNA profiles in the tumors [106]. Moreover, Kan et al. [124] revealed that individual levels of miR-200a, miR-200b and miR-200c normalized to serum volume and miR-103 were significantly higher in the sera of the serous ovarian cancer cohort. A multivariate model combining miR-200b and miR-200c readings showed good predictive power to discriminate serous ovarian cancer patients and healthy controls. Kapetanakis et al. showed that plasma miR-200b levels were significantly higher in ovarian cancer patients than in patients with benign tumors, and suggested that miR-200b can be a complementary biomarker to CA125 [125]. Hence, miR-200s may be good diagnostic biomarkers for biopsy screening.

For ovarian cancer prognosis, several studies have studied the association between circulating miR-200 levels and disease prognosis. In a study of serum miR-200 levels in 74 ovarian cancer patients, both miR-200c and miR-141 were significantly elevated in cancer sera than in healthy control [126]. However, the two miR-200 members showed discordant prognostic values. Only high miR-200c levels and low miR-141 levels were significantly associated with a good survival rate [126]. In a study to compare pre/post-treatment variations of plasma miR-200b levels in ovarian cancer, patients with reducing plasma miR-200b levels showed a longer progression-free survival than those patients with increasing miR-200b levels, and the patients with increasing miR-200b levels had a higher risk of disease progression [125]. Taken together, the current studies have shown promising results for serum or plasma miR-200 levels as diagnostic and prognostic biomarkers. Further large-scale studies are warranted to study the potential clinical utility of miR-200s in the blood and other body fluids such as urine and saliva.

As functional studies have shown that miRNA dysregulation is causal in many human cancers, several miRNA-targeted therapeutics employing miRNA mimics or miRNA-targeting molecules (antimiRs) have reached clinical development [127]. Cortez et al. have shown that systemic delivery of miR-200c to the cancer cells in a lung cancer xenograft model could increase cellular radiosensitivity by modulation of miR-200c on oxidative stress response genes [128]. A similar proposal has been suggested for ovarian cancer treatment based on suppressive function of miR-200s on EMT in cancer metastasis [129]. However, in our view, miR-200 function in epithelial cell identity and maintenance has a positive effect on ovarian cancer cell growth and collective movement in metastasis. There are too many conflicting results and discordant data from different studies, which are subject to a variety of factors including tumor type, tumor stage, cellular ROS content, and availability of interacting partners. We suggest that a preliminary study using appropriate animal models with standardized cell context and other variables should be cautiously performed to establish the role of miR-200s in chemoresponse as a new avenue for therapeutic intervention.

## 3. Conclusions

The miR-200 family is a master regulator of EMT in epithelial ovarian cancer. Despite extensive investigation of its role and function in tumor initiation, progression, metastasis, and chemoresponse, there are still contradictory views in determining whether this miRNA family is beneficial or a hindrance to treat the disease. Expression of miR-200s is important for cancer cell growth and local dissemination. On the other hand, the presence of miR-200s may suppress hematogenous metastasis and angiogenesis, and may or may not influence the chemoresponse of the cancer cells. Spatial and temporal presence or absence of the miRNA and putative partners such as HuR may play a significant role [116]. The current results of miR-200s as cancer diagnostic and prognostic biomarkers are encouraging. More studies with standardized controls are recommended to establish whether this miRNA family plays a role in the therapeutics of ovarian cancer.

## Figures and Tables

**Table 1:** Function of miR-200s in different organs and tissue types.

Types of Organ/Tissue	Models/Cell Lines	Member(s) Involved	Function	Pathways Involved	Ref.
Taste bud	Zebra fish	miR-200a, miR-200b, miR-429	Taste bud formation and in particular for Calb2b(+) cell formation	Notch signaling acted upstream of miR-200s	[12]
Forebrain	Mouse	miR-200a, miR-200b, miR-429	Neurogenesis	miR-200s mediated ZEB2 for late stage neuronal maturation in postnatal olfactory bulb neurogenesis	[8]
Kidney	Mouse	miR-200a, miR-200b, miR-429	Differentiation of podocytes	miR-200s potentially promoted podocyte differentiation through repression of RSAD2 expression	[11]
Neural stem cell	Rat	miR-200a, miR-200b	Differentiation of neural stem cell	Over-expression of miR-200s induced neurite formation in PC12 cells and regulated neuronal markers in favor of differentiation.	[10]
Forebrain	Zebra fish	miR-200a, miR-200b, miR-141, miR-429	Differentiation of neuron	miR-200s negatively controlled Foxg1 mRNA, a fork-head transcription factor essential for development of the olfactory epithelium and of the forebrain	[9]
Fat cell	Drosophila	miR-8 (homolog of miR-200)/miR-200c/miR-141	Fat cell growth and organismal growth	miR-200 specifically modulated PI3K-AKT-FOXO signaling	[7]
Uterus	Human	miR-200a, miR-200c, miR-141, miR-429	Decidualization	miR-200s were downregulated during decidualization. Potential targets was included in the cell cycle pathway. The most represented pathway was the ErbB signaling	[13,14]
Kidney	Mouse	miR200b, miR-200c, miR-429	Knockdown inhibited tubulogenesis and produced cyst-like structure	miR-200b/c/429 induced post-transcriptional repression of polycystin 1 (PKD1) through two conserved binding sites in the 3′-untranslated regions of PKD1	[16]
Testis	Yellow catfish	All five members	Down-regulation of miR-141 and 429 during testis development.	miR-200s were up-regulated when testis were treated with high dose of estrogen that would impair testis development and cell proliferation	[17]

**Table 2:** Other functions of miR-200s in ovarian and gynecologic cancers.

Processes	Cell Lines	Member(s) Involved	Pathways Involved/Function	Ref.
Angiogenesis	Ishikawa cells	miR-200c	Gain of function of miR-200c in Ishikawa cells repressed ZEBs, as well as VEGFA, FLT1, IKKβ, and KLF9 expression at transcriptional and translational levels	[92]
Angiogenesis	Hey8A	miR-200c	miR-200c decreased IL8 and CXCL1 expressions	[94]
Exosome Production	SKOV3, OVCAR3	miR-200a, miR-200b, miR-200c	May be related to invasiveness	[107]
Chemoresistance	SKOV, HEY, OVCA1847	miR-200c	Negatively regulate TUBB3, which overexpression results in resistance to taxane	[116]
Chemoresistance	OVCAR-3, MES-OV	miR-200c, miR-141	Lentiviral transfection of inhibitors of miR-200c or miR-141 in parental OVCAR-3 rendered the cells resistant to paclitaxel and carboplatin	[117]

## Abbreviations

3′-UTR	3′-Untranslated region
3D	Three-dimensional
AGO2	Argonaute 2
AKR1C1	Aldo-keto reductases 1C1
AKRIC4	Aldo-keto reductases 1C4
AKT	Protein kinase B
ALDHA3	Aldehyde dehydrogenase 1A3
AntimiRs	miRNA-targeting molecules
BMI1	Polycomb group RING finger protein 4
CA125	Cancer antigen 125
CDH1	E-cadherin
CXCL1	Chemokine (C-X-C motif) ligand 1
ECM	Extracellular matrix
EGFR	Epidermal growth factor receptor
EMT	Epithelial-mesenchymal transition
ErbB3	Erb-B2 receptor tyrosine kinase 3
ERK	Extracellular signal–regulated kinases
ERRa	Estrogen-related receptor alpha
FLT1	Fms related tyrosine kinase 1
FOG2	Friend of GATA 2
Foxg1	Forkhead box G1
FOXO	Forkhead box O3
FTE	Fallopian tube epithelial
GRHL2	Grainhead-like 2
HGSOC	High-grade serous ovarian carcinoma
Hnf-1β	Hepatocyte nuclear factors 1β
hnRNPA2B1	SUMOylated heterogeneous nuclear ribonucleoprotein A2B1
HuR	Human antigen R
IKKβ	I-κB kinase β
IL8	Interleukin 8
IRS-1	Insulin receptor substrate 1
KDR	VEGF receptor 2
KLF9	Kruppel like factor 9
KRAS	V-Ki-ras2 Kirsten rat sarcoma viral oncogene homolog
MAPK	Mitogen-activated protein kinase
MEK	Mitogen-activated protein kinase kinase
MET	Mesenchymal-epithelial transition
miR-200s	MicroRNA-200 family members
miRNAs	MicroRNAs
mRNA	Messenger RNA
MTHFD2	Methylene tetrahydrofolate reductase 2
NCI60	A panel of 60 diverse human cancer cell lines
NRG1	Neuregulin
OSE	Normal ovarian surface epithelial
PI3K	Phosphatidylinositol-4,5-bisphosphate 3-kinase
PKD1	Polycystin I
RISC	RNA-induced silencing complex
ROCK	Rho-associated protein kinase
ROS	Reactive oxygen species
RRM2	Ribonucleotide reductase subunit 2
RSAD2	Radical *S*-adenosyl methionine domain containing 2
RT-PCR	Quantitative reverse transcription-polymerase chain reaction
S6K	Ribosomal protein S6 kinase
SCD	Stearoyl-CoA desaturase
SIRT1	Sirtuin 1
SMAD2	Similar to mothers against decapentaplegic family member 2
SMAD3	Similar to mothers against decapentaplegic family member 3
STIC	Serous tubal intraepithelial carcinoma
TCGA	The recent Cancer Genome Atlas
TGF-β	Transforming growth factor-β
TUBB3	Class III β-tubulin gene
TXNDC12	Thioredoxin domain containing 12
VEGF	Vascular-endothelial-growth factor
ZEB1	Zinc finger E-box-binding homeobox 1
ZEB2	Zinc finger E-box-binding homeobox 2
